# Blood donation candidates who are deferred in the State of Roraima, Brazilian Amazon: reasons for unsuitability and seroprevalence of transfusion-transmitted pathogens

**DOI:** 10.1590/0074-02760250320

**Published:** 2026-10-30

**Authors:** Daniela Ribeiro Roque, Fabiana Granja, Filipe Anibal Carvalho-Costa

**Affiliations:** 1 Fundação Oswaldo Cruz-Fiocruz, Instituto Oswaldo Cruz, Laboratório de Epidemiologia e Sistemática Molecular, Rio de Janeiro, RJ, Brasil; 2 Laboratório Central de Saúde Pública de Roraima, Setor de Imunologia/Parasitologia, Boa Vista, RR, Brasil; 3 Universidade Federal de Roraima, Centro de Estudos da Biodiversidade, Laboratório de Biologia Molecular, Boa Vista, RR, Brasil

**Keywords:** transfusion-transmitted diseases, seroprevalence, Roraima, blood donation

## Abstract

**BACKGROUND:**

Blood donation candidates who are deferred are not targeted by any health policy.

**OBJECTIVES:**

To describe sociodemographic characteristics of unsuitable blood donation candidates, the causes of unsuitability and compare the prevalence rates of transfusion-transmitted infections in deferred and eligible blood donors.

**METHODS:**

A cross-sectional survey was conducted to obtain sociodemographic data and blood samples; 8,159 blood donation candidates were screened (21.1% rejected, 78.9% approved). A sample of 212 deferred candidates was established.

**FINDINGS:**

Most rejected candidates were female, young adults, with high school or university education, self-declared white, born in the North region and single. Main reasons for unsuitability were low haemoglobin concentration, potential exposure to malaria and risky sexual behaviour. The comparison of seropositivity rates in deferred and eligible blood donation candidates generated the following prevalence ratios (PR) and respective 95% confidence intervals (CI): human immunodeficiency virus (HIV), PR = 6.75 (1.46 - 31.05), p = 0.046; syphilis, PR = 1.47 (0.46 - 4.64), p = 0.463; anti-HBc, PR = 2.84 (1.24 - 6.50); p = 0.023; anti-HCV, PR = 4.67 (1.06 - 20.58), p = 0,080; Chagas disease, PR = 60.75 (5.53 - 667.43), p = 0.003.

**MAIN CONCLUSIONS:**

Treating anaemia in women and controlling malaria in Roraima will reduce the pool of rejected blood donation candidates in the State.

In Brazil, blood donation is strictly voluntary and non-remunerated, and blood collection is carried out through a network of public blood banks managed by the federative states within the Unified Health System (Sistema Único de Saúde — SUS), with a minor contribution from a state-regulated private sector.^(1,2)^ At blood banks, donated blood is tested for the presence of antibodies against blood antigens of transfusion importance and submitted to serological tests to detect the most prevalent transfusion-transmitted pathogens [human immunodeficiency virus (HIV), human T-cell lymphotropic virus type I/II (HTLV I/II), hepatitis B virus (HBV), hepatitis C virus (HCV), Chagas disease (CD), and syphilis] in addition to undergoing nucleic acid tests (NATs) to detect HIV, HBV, HCV, and malaria.^(1,2,3)^ After these analyses, donated blood is processed to produce red blood cell concentrate, plasma and platelet concentrate. Other blood products include human albumin and immunoglobulins.

Since the 1980s, Brazil has prohibited paid blood donation, in addition to testing donated blood for the diseases mentioned above.^(1,2)^ In the pre-testing era, the presence of blood-borne diseases among the population of paid donors has led to the occurrence of several cases of transfusion-transmitted infections.^(4,5,6)^

The window period of serological tests allow blood contaminated with transfusion-transmitted pathogens to present a negative test.^(7)^ This requires questionnaires on sexual behaviour and investigation of other possible exposures to pathogens in the population of candidates for blood donation, which may characterize the potential donor as deferred.^(1,2,8)^ Candidates deferred for donation are dismissed without performing serological tests, so the frequency of infection by blood-borne pathogens in this population is unknown. Knowing the seropositivity rates for transfusion-transmitted pathogens among deferred candidates for donation could improve donor selection strategies. NATs are also capable of detecting infections by these agents before seroconversion, reducing the window period and increasing the safety of transfusions.^(9)^

Among the conditions that lead to ineligibility to donate blood in Brazil are factors related to sexual behaviour, chronic diseases, as well as simpler conditions such as low blood haemoglobin concentration, use of some medications and weight below 50 kg. There is a wide range of conditions that make donation not possible, both temporarily and permanently, which are described in the laws that regulates hemotherapy in Brazil.^(1,2,10)^ In regions where malaria is endemic, such as the states belonging to the Brazilian Amazon, many blood donation candidates may be deferred based on the criterion of having visited areas of active malaria transmission, which reduces substantially the pool of potential donors in the population.^(8)^ In this context, the incidence of malaria in Roraima is increasing, strongly impacted by the interaction between gold mining and indigenous populations.^(11)^

In recent years, the State of Roraima has been receiving massive waves of migration from Venezuela. Venezuelans come to Brazil in search of better living and working conditions, Roraima being the gateway and destination for a large proportion of migrants. In a context of poverty and unemployment, some Venezuelan women have found prostitution to be the only way to survive in Brazil.^(12)^ The new sex trade networks in the State capital, Boa Vista, could potentially amplify the transmission of sexual transmitted infections (STIs)^(13)^ and increase the proportion of blood donation candidates rejected in the State of Roraima. It should be emphasized that, regardless of this migratory process, the incidence of STIs, including syphilis, has increased substantially in Brazil.^(14)^ In addition, the movement of people across the Brazil-Venezuela border, especially in Indian Lands, harms malaria control.^(15)^

The present study aimed to describe a sample of donation candidates deferred by the Haemotherapy and Haematology Centre of Roraima (Haemoraima), including the reasons for unsuitability and frequency of transfusion-transmitted infections.

## MATERIALS AND METHODS

*Setting* - The State of Roraima, in the extreme north of Brazil, is in the Brazilian Amazon, bordering Venezuela and Guyana. It is virtually isolated by road and can be accessed by air. The State has 636,707 inhabitants distributed over 223.505 Km^2^, which gives it a very low population density of 2.85 inhabitants/Km^2^; 46% of its territory is represented by Indian Lands, mainly the Yanomami Indian Land and the Raposa Serra do Sol Indian Land. The human development index is 0.699 and the capital, Boa Vista (2º49′12″ N, 60º40′19″W), has 413,486 inhabitants.^(16)^

*Study design, sampling, recruitment strategy and statistical analysis* - A cross-sectional survey was conducted with a sample of deferred blood donation candidates at the Haemoraima, to obtain sociodemographic data and blood samples for serological testing for HIV, *Treponema* *pallidum* (syphilis), HBV and HCV, HTLV, and *Trypanosoma* *cruzi* infection (CD). During the period from June to October 2024, 8,159 donation candidates were screened at Haemoraima, of which 1,719 (21.1%) were rejected for various reasons and 6,440 (78.9%) were approved. A random sample of 212 deferred candidates was established, based on the following parameters: expected frequency = 10%, margin of error = 2.5% and confidence level = 80%. Prevalence ratios and their respective 95% confidence intervals (CIs) were calculated based on the prevalence of transfusion-transmitted infections among deferred and eligible donation candidates. The statistical significance of the associations was verified using Fisher’s exact test, with a significance level set at p < 0.05.

*Classification of donation candidates as eligible and deferred* - Regarding social and behavioural characteristics, candidates for blood donation are considered deferred if they have risky sexual behaviour (*e.g.*, have multiple sexual partners, have had non-habitual sexual intercourse without using a condom, have had sexual intercourse with sex workers, with injectable drug addicts, with people who have had hepatitis, with people living with HIV, with transfused persons or with ex-convicts), reside in or have visited a malaria-endemic region less than one month ago, use marijuana or have had a tattoo, piercing, permanent makeup or acupuncture less than six months ago. Medical conditions that prevent donation include low blood haemoglobin concentration/haemorrhage, high or low blood pressure, weight less than 50 kg, serious illness less than one (1) month, flu-like illness, major surgery in the last six months, dental treatment in the last 72 h, minor surgery in the last three months or heart rate above 110 bpm. Have received vaccines against rabies, meningitis, influenza, tuberculosis [Bacillus Calmette-Guérin (BCG)], measles-mumps-rubella or measles alone in the last four weeks also prevents donation. Among previous infectious diseases that make the candidate unsuitable are toxoplasmosis, malaria, syphilis, CD, HIV/acquired immunodeficiency syndrome (AIDS), hepatitis, dengue, rubella, brucellosis and mononucleosis. Other conditions include have had received blood/components transfusion less than one year, chronic kidney disease/renal replacement therapy, diabetes, hyper or hypothyroidism, autoimmune diseases, haematological diseases/haemoglobinopathies, alcoholism and use of antibiotics in the last five days. Blood haemoglobin concentration was measured in the screening of potential donors with the non-invasive OrSense Haemoglobin Analyser (OrSense Ltd, Tel Aviv, Israel).

*Serological tests* - The serological tests performed on the sample of deferred candidates and on the population of eligible donors were as follows: ARCHITECT HIV Ag/Ab Combo [a chemiluminescent microparticle immunoassay (CMIA), ARCHITECT anti-HCV (CMIA), ARCHITECT Syphilis TP (CMIA), ARCHITECT rHTLV-I/II (a CMIA test that screens for proviral DNA), ARCHITECT Chagas (a CMIA for the qualitative detection of antibodies to *T. cruzi*), ARCHITECT Anti-HBc II, and ARCHITECT HBsAg (all provided by Abbott Laboratories, Lake Bluff, Illinois, USA)].

*Ethics* - The study was approved by the Ethics Committee on Research of the Federal University of Roraima, protocol CAAE 75834223.9.0000.5302.

## RESULTS

*Sociodemographic characteristics and reasons for ineligibility to donate* - Table I shows that most rejected blood donation candidates were female, with a predominance of young adults with high school or university education and self-declared white, born in the North region and single. As presented in Table II, the main reasons for deferred for blood donation were low blood haemoglobin concentration, potential exposure to malaria and risky sexual behaviour, in addition to the presence of tattoos and piercings.

**TABLE I:** Sociodemographic characteristics of deferred blood donors in the Haemotherapy and Haematology Centre of Roraima (Haemoraima), Boa Vista, Roraima, 2024

Gender	N	%
Female	136	64
Male	76	36
Age group (years)		
18-19	7	3.3
20-29	73	34.4
30-39	69	32.6
40-49	44	20.8
50-59	18	8.5
60-69	1	0.5
Education		
Elementary	9	4.3
High school	96	45.3
University	96	45.3
Unknown	11	5.2
Ethnicity		
Yellow	17	8.0
White	172	81.1
Indian	2	0.9
Brown	5	2.4
Black	9	4.3
Unknown	7	3.3
Brazilian region where was born		
North	152	71.7
Northeast	45	21.2
South	1	0.5
Southeast	8	3.8
Central-West	0	0
Stranger	4	1.9
Unknown	2	0.9
Marital status		
Single	131	61.8
Married	43	20.3
Other	24	11.3
Divorced	6	2.8
Unknown	8	3.8

**TABLE II:** Causes of non-suitability for blood donation in candidates for donation in the Haemotherapy and Haematology Centre of Roraima (Haemoraima), Boa Vista, Roraima, 2024

Condition impairing blood donation	Male	Female	N	%
Low blood haemoglobin concentration	2	57	59	27,8
Potential exposition to malaria	22	19	41	19,3
Sexual behaviour	21	17	38	17,9
Not listed	4	12	16	7,6
Tattoo/piercings	0	11	11	5,2
Use of medicines	5	4	9	4,3
Vaccines	6	1	7	3,3
Hyper- or hypotension	3	4	7	3,3
Any definitive condition	5	0	5	2,4
Autoimmune diseases	2	2	4	1,9
Thyroid diseases	1	2	3	1,4
Flu	1	2	3	1,4
Low body weight	0	2	2	0,9
Haematological disease	2	0	2	0,9
Hepatitis after 10 years-old	2	0	2	0,9
Surgery	0	1	1	0,5
Tachycardia	0	1	1	0,5
Transfused less than on year	0	1	1	0,5
Total	76	136	212	100

*Prevalence of transfusion-transmitted infections among eligible and deferred blood donation candidates* - As presented in Table III, the sample of rejected candidates showed an overall seropositivity rate, for any pathogen, significantly higher than the population of donors approved in the social and medical questionnaire. Analysing the pathogens individually, the seropositivity rates and the number of seropositive individuals among refused candidates were 0.94% (n = 2) for anti-HIV, 1.4% (n = 3) for syphilis, 2.8% (n = 6) for anti-HBC, 0% (n = 0) for HBsAg, 0.94% (n = 2) for anti-HCV, 0% (n = 0) for HTLV I/II and 0.94% (n = 2) for CD. Among the 6,440 eligible donors, the seropositivity rates and the number of seropositive individuals were 0.14% (n = 9) for anti-HIV, 0.96% (n = 62) for syphilis, 0.99% (n = 64) for anti-HBC, 0.09% (n = 6) for HBsAg, 0.2% (n = 13) for anti-HCV, 0.03% (n = 2) for HTLV I/II and 0.02% (n =1) for CD. The comparison of seropositivity rates in deferred and eligible blood donors generated the following prevalence ratios (PR) and respective 95% CI: HIV, PR = 6.75 (1.46 - 31.05), p = 0.046; syphilis, PR = 1.47 (0.46 - 4.64), p = 0.463; anti-HBC, PR = 2.84 (1.24 - 6.50); p = 0.023; anti-HCV, PR = 4.67 (1.06 - 20.58), p = 0,080; CD, PR = 60.75 (5.53 - 667.43), p = 0.003. For HBsAg and HTLV I/II the PRs were undefined.

**TABLE III:** Comparison of seropositivity rates for different transfusion-transmitted pathogens in suitable and unsuitable blood donors in the Haemotherapy and Haematology Centre of Roraima (Haemoraima), Boa Vista, Roraima, 2024

	Eligible (n = 6,440)			Deferred (n = 212)			
	Positive (n)	Positive (%)		Positive (n)	Positive (%)	PR (95% CI)	p-value^*^
Chagas disease	1	0.02		2	0.94	60.75 (5.53 - 667.43)	0.003
HTLV I/II	2	0.03		0	0	Undefined	NP
HCV	13	0.2		2	0.94	4.67 (1.06 - 20.58)	0.080
Syphilis	62	0.96		3	1.4	1.47 (0.46 - 4.64)	0.463
HBsAg	6	0.09		0	0	Undefined	NP
HIV	9	0.14		2	0.94	6.75 (1.46 - 31.05)	0.046
Anti-HBc	64	0.99		6	2.8	2.84 (1.24 - 6.50)	0.023
Any test	157	2.4%		15	7.1%	2.90 (1.73 - 4.84)	< 0.001

PR: prevalence ratio; CI: confidence interval;

* assessed through Fisher’s Exact Test.

## DISCUSSION

The present study aimed to characterise blood donation candidates deferred by a blood bank in the Brazilian Amazon. The sociodemographic characteristics of the rejected candidates indicate a predominance of women, which can be explained by the fact that the main cause of unsuitability was low blood haemoglobin concentration. Regarding age, it was demonstrated that many unsuitable candidates are young adults, a fact related to the frequency of some characteristics considered incompatible with donation, such as risky sexual behaviour and the presence of tattoos and piercings. The age distribution of unsuitable candidates can be related to the finding that most of them have a good level of education, with high school or university education and are single.

This study demonstrated that the three main causes of ineligibility to donate blood in the State of Roraima are low blood haemoglobin concentration, potential exposure to malaria and risky sexual behaviour. Anaemia, which for the purposes of donation candidate refusal is characterised by a haemoglobin concentration of less than 12.5 g/dL for women and 13 g/dL for men, is the main cause of ineligibility to donate blood globally.^(17)^ A previous study conducted at Haemoraima demonstrated that the frequency of ineligibility due to low haemoglobin concentration was 23.9%^(18)^ and, therefore, this situation persists to the present day. This finding is also related to the high prevalence of iron deficiency in the population, and it should also be considered that in women, menstrual losses associated with abnormal uterine bleeding are an important cause of anaemia. Recent studies have documented a higher prevalence of low haemoglobin concentration in female blood donation candidates when compared to male candidates.^(19,20)^ In the present study, one-fifth of donation candidates were rejected due to potential exposure to malaria. In a previous study carried out in Roraima five years ago, a different pattern of causes of deferral for donation was observed, in which potential exposure to malaria accounted for only 1.5% of deferral.^(18)^ The incidence of malaria in Roraima has increased, influenced by the vulnerability associated with the interaction of gold mining with Indian societies,^(21)^ so that all municipalities in the state currently have active transmission^(22)^ and it should also be considered that a proportion of the reported cases are imported from Venezuela.^(23)^ In Roraima, a large proportion of the territory is part of the Yanomami and Raposa Serra do Sol Indian Lands, which have extensive borders with Venezuela and Guyana. The expansion of illegal gold mining in these regions is related to the increasing incidence of malaria in the State. Of the 15 municipalities in the State of Roraima, seven were classified in 2023 as high risk for malaria (incidence > 50 cases/1000 inhabitants/year).^(22)^ Having visited any of these municipalities in the 30 days prior to donation is a reason for ineligibility, so malaria has played an increasing role in refusing blood donation candidates in Roraima. It is important to note that the blood of all approved donors is also subjected to a malaria NAT test.^(24)^ The third main reason for refusal to donate was risky sexual behaviour. In the previous study carried out at Haemoraima, this was the second most frequent cause, ahead of malaria.^(18)^ New patterns of sexual behaviour have been observed with an increase in the number of sexual partners, and this may be related to the fact that many rejected candidates are young people.^(25,26)^

In the present study, the fact that risky sexual behaviour was identified in a group of unfit candidates could be related to the relatively higher seroprevalence rates for some transfusion-transmitted pathogens, which are also STIs, such as HIV and HBV. However, although one of the two HIV-positive unsuitable candidates was rejected for risky sexual behaviour, among the six unsuitable donors who were positive for hepatitis B, only one was in this rejection category, so the other five were rejected for other reasons (three had been in an area of active malaria transmission). The study’s main limitation was the need to sample deferred candidates, so that wide confidence intervals were generated when the prevalence rates of transfusion-transmitted diseases were compared. In this sense, although the HIV, HBV, and CD seropositivity rates were significantly higher among rejected candidates, the wide confidence intervals mean that this data needs to be interpreted with caution. Concerning HCV, there was also a trend towards greater seropositivity among refused donation candidates, although without statistical significance at the 5% level. We did not have access to information on whether donor candidates were first-time or repeat donors, although we know that only a minority donate blood repeatedly. However, because our unit of analysis was the donor rather than each donation visit, we believe that the lack of stratification by donor status (first-time vs. repeat donors) did not introduce bias into the comparative analysis of infection frequency across different pathogens among eligible and deferred candidates.

In the present study, the seroprevalence of CD was higher among rejected blood donation candidates when compared to approved donors. One of these two CD-seropositive candidates was deferred because he had hepatitis after 10 years old and the other because he had malaria in the last 12 months, not having been deferred because they presented CD previously. It is possible that serological cross-reactions with leishmaniasis occur and that the greater exposure to malaria among rejected donation candidates is associated to greater exposure to leishmaniasis. The identification of false-positive individuals for CD due to serological cross-reactions with *Leishmania* antigens has been a cause for concern, especially in areas where both diseases are endemic.^(27)^ It should be noted that, although 95% (4,169/4,404) of reports of acute CD in Brazil between 2008 and 2023 came from the Amazon region, only five cases were reported in the State of Roraima.

In conclusion, the current policy of classifying potential blood donation candidates as unfit contributes to the safety of blood transfusion. Female candidates with haemoglobin levels close to the lower limit of normal or low could have their clinical condition clarified and be advised to seek a health unity to have the cause of this clinical condition evaluated, receive treatment and become eligible donors in the future.^(28)^ Better control of malaria in the State of Roraima will also contribute to reducing the rate of ineligibility for blood donation.

## Data Availability

The contents underlying the research text are included in the manuscript.
