# Supplementary material for: Blood donation candidates who are deferred in the State of Roraima, Brazilian Amazon: reasons for unsuitability and seroprevalence of transfusion-transmitted pathogens
Source: Mem Inst Oswaldo Cruz. 2026 Oct 30;121(Suppl 1):e250320. doi: 10.1590/0074-02760250320 (PMC13722438; doi:10.1590/0074-02760250320)
Supplement: Peer review history

# Reviewer report: FIRST REVIEW ROUND - REVIEWERS’ COMMENTS

## REVIEWER #1

In Brazil, blood donation is mandatorily voluntary and unpaid, and collection is done exclusively through a system of public blood banks managed by federative states, within the Unified Health System. (1,2). That is not correct, as the private sector also plays a role in the blood collection Brazilian sustem – Please correct the phrase

In the phrase “donated blood is fractionated to produce”, change to “donated blood is processed to produce”

Page 7 – Correct “Among previous infectious diseases that make de donor unsuitable are” to “ Among previous infectious diseases that make the donor unsuitable are”

Overall, there is a semantic issue between candidates and donors, since candidates are those individuals who were still under clinical interview before donation; if accepted, then his/her condition changes from candidate to donor. Otherwise, if the clinical interview detects something that prevents a blood donation, then this individual will remain as a deferred (or rejected) candidate, but not a donor. Furthermore, after donation, the donor blood sample will be subjected to a series of tests, where after all results, the blood donation will be considered as approved for transfusion or rejected. Thus, authors should rephrase the paragraphs When there are comparisons between a deferred/rejected candidate versus accepted/rejected donor.

There is no comparison between first time versus repeated donors, either in the pre-donation (clinical) stage or after donation. This is of paramount importance and all statistical data should also stratify first time repeated donation. Even if the percentage of repeated donors is low, it must be discussed in the study

Overall, there issues must be addressed and corrected by authors, before this paper can be re-considered for reviewing.

## AUTHORS’ RESPONSE TO THE REVIEWERS

Specific answers for reviewers

We thank the reviewer for their careful assessment, which has greatly improved the manuscript. All comments have been incorporated into the text. The changes are highlighted in yellow.

Reviewer: 1

In Brazil, blood donation is mandatorily voluntary and unpaid, and collection is done exclusively through a system of public blood banks managed by federative states, within the Unified Health System. (1,2). That is not correct, as the private sector also plays a role in the blood collection Brazilian sustem - Please correct the phrase

Answer: We corrected this information.

In the phrase “donated blood is fractionated to produce” , change to “donated blood is processed to produce”

Answer: We changed fractioned for processed.

Page 7 - Correct “Among previous infectious diseases that make de donor unsuitable are” to “ Among previous infectious diseases that make the donor unsuitable are”

Answer: We corrected this.

Overall, there is a semantic issue between candidates and donors, since candidates are those individuals who were still under clinical interview before donation; if accepted, then his/her condition changes from candidate to donor. Otherwise, if the clinical interview detects something that prevents a blood donation, then this individual will remain as a deferred (or rejected) candidate, but not a donor. Furthermore, after donation, the donor blood sample will be subjected to a series of tests, where after all results, the blood donation will be considered as approved for transfusion or rejected. Thus, authors should rephrase the paragraphs When there are comparisons between a deferred/rejected candidate versus accepted/rejected donor.

Answer: We corrected this throughout the text.

There is no comparison between first time versus repeated donors, either in the pre-donation (clinical) stage or after donation. This is of paramount importance and all statistical data should also stratify first time repeated donation. Even if the percentage of repeated donors is low, it must be discussed in the study

Answer: We fully agree that this issue should be clearly justified. We added the following sentence to de Discussion. “*We did not have access to information on whether donor candidates were first-time or repeat donors, although we know that a minority donate blood repeatedly. However, because our unit of analysis was the donor rather than each donation visit, we believe that the lack of stratification by donor status (first-time vs. repeat donors) did not introduce bias into the comparative analysis of infection frequency across different pathogens among eligible and deferred candidates*.”

# Reviewer report: REVIEWERS’ COMMENTS

## REVIEWER #1

No comments.
